# Clinical characteristics and outcomes of acute liver failure in neonates: a retrospective cohort in China

**DOI:** 10.1007/s00431-024-05567-7

**Published:** 2024-04-30

**Authors:** Suhua Xu, Peng Zhang, Mengmeng Ge, Yuanyuan Shan, Guoqiang Cheng

**Affiliations:** 1 Department of Neonatology, Shanghai Children’s Hospital, School of Medicine, Shanghai Jiao Tong University, Shanghai, China; 2 Department of Neonatology, Children’s Hospital of Fudan University, National Children’s Medical Center, Shanghai, China; 3 Fujian Key Laboratory of Neonatal Diseases, Xiamen Children’s Hospital, Xiamen, China

**Keywords:** Liver failure, Hepatology, Neonate, Perinatal asphyxia, Neonatal hemochromatosis

## Abstract

Neonatal acute liver failure (NALF), as a rare disease with high mortality, has limited relevant literature reports in China. We attempted to analyze a NALF cohort to improve the prognosis of this disease. We included all patients diagnosed with NALF at our institution between 2016 and 2021 and retrospectively reviewed their electronic records. NALF was defined as an INR ≥ 2.0 due to liver disease 28 days after birth. Comparisons were made according to etiology and outcome. The Kaplan-Meier method was used to estimate survival. Fifty-eight patients were included in this study. Etiologies included hypoxic/ischemic injury (29.3%), infection (27.6%), gestational alloimmune liver disease with neonatal hemochromatosis (GALD-NH) (10.3%), inherited metabolic diseases (5.2%), hemophagocytic lymphohistiocytosis (1.7%), other etiologies (12.1%), and unidentified causes (13.8%). Enteroviruses constituted 87.5% of the viral infections, whereas herpes simplex virus accounted for no infections. The median INR was significantly lower in the infection group than in the GALD-NH group (*P* < 0.05 for multiple comparisons). At the last follow-up, none of the patients had undergone liver transplantation, and the overall mortality rate was 50%. Liver function completely recovered in 31% of the patients, all of whom survived. The overall median survival time was 48 days; 26 days for hypoxic/ischemic injury and 43 days for GALD-NH. The incidence of cholestasis was significantly greater among surviving patients (*P* = 0.018).

*   Conclusion*: Hypoxic/ischemic injury and infection are the predominant etiologies of NALF in China. The overall prognosis of NALF is poor, but its short-term prognosis is determined by the etiology.

**Table:** 

**What is Known:** *• Neonatal acute liver failure (NALF) is a rare disorder with limited cohort studies, especially in China.* *• Gestational alloimmune liver disease, viral infections (especially herpes simplex virus), metabolic diseases and ischemic insults are common etiologies of NALF, which are significantly different from other populations.* *• There are no reliable biochemical markers to predict the outcome of NALF.*	
**What is New:** *• In this first report on a Chinese NALF cohort, we demonstrate that hypoxic/ischemic injury and infection (excluding herpes simplex virus) are the predominant etiologies of NALF.* *• The overall prognosis of NALF is poor, and its etiology determines the short-term outcome.*

**What is Known:**

*• Neonatal acute liver failure (NALF) is a rare disorder with limited cohort studies, especially in China.*

*• Gestational alloimmune liver disease, viral infections (especially herpes simplex virus), metabolic diseases and ischemic insults are common etiologies of NALF, which are significantly different from other populations.*

*• There are no reliable biochemical markers to predict the outcome of NALF.*

**What is New:**

*• In this first report on a Chinese NALF cohort, we demonstrate that hypoxic/ischemic injury and infection (excluding herpes simplex virus) are the predominant etiologies of NALF.*

*• The overall prognosis of NALF is poor, and its etiology determines the short-term outcome.*

## Introduction

Acute liver failure (ALF) is an altered state characterized by a sudden loss of liver function without any previous liver disease, and the incidence and etiology of this disease vary according to age group and geographic region [1, 2]. Neonatal acute liver failure (NALF) is defined as liver synthetic dysfunction with an international normalized ratio (INR) ≥ 2.0 in the first 4 weeks of life [1], including neonatal cirrhosis due to liver damage in the fetus [3].

The etiological spectrum and clinical features of NALF, as a rare disease with high mortality, are significantly different from other populations [1, 3]. However, most of the published reports of ALF include pediatric and neonatal populations [2, 4, 5], with limited data for neonates [6–9]. And there are limited data on Chinese newborns with ALF. Therefore, we retrospectively analyzed the etiologies, clinical features, therapeutic treatments and outcomes of patients diagnosed with NALF in China. The results of this study could contribute to early diagnosis and effective treatment to improve the outcomes of NALF patients.

## Methods

### Study design and patients

This was a single-center retrospective study conducted in a large tertiary pediatric hospital. We reviewed all coagulation tests performed in the Neonatology Department of the Children’s Hospital of Fudan University between January 1, 2016, and December 31, 2021, to identify patients that fit the NALF definition via a ‘catch-all’ approach. The inclusion criteria in our study were inpatients (term infants aged ≤ 28 days and preterm infants with a postmenstrual age less than 44 weeks) with an INR ≥ 2.0. The exclusion criteria were as follows: no underlying liver synthetic dysfunction presenting as transient consumptive coagulopathy, vitamin K responsive coagulopathy, congenital coagulation factor deficiency and neoplasms.

### Definitions and diagnostic criteria

All patients diagnosed with NALF in our institution underwent extensive work-up to determine the causes. Infection was determined by blood, urine and fecal cultures, serological and molecular methods (polymerase chain reaction or macrogenomics). Neonatal hemochromatosis (NH) was diagnosed when extrahepatic iron overload was confirmed on salivary gland biopsies or magnetic resonance imaging (MRI), and classified as gestational alloimmune liver disease with neonatal hemochromatosis (GALD-NH) if no other etiology of NH was identified. Inherited metabolic diseases (IMDs) were diagnosed by measuring the relevant metabolites in the urine or plasma and then further confirmed by genetic testing. When mitochondrial disorders were suspected due to clinical history and examinations, we performed respiratory chain enzymes and mitochondrial DNA (mtDNA) copy number in liver and/or muscle, if feasible. Furthermore, we performed abdominal ultrasonography and/or MRI to find portosystemic shunts, hemangiomas, liver and/or biliary lesions. Our diagnostic protocol in NALF cases also included measurement of ferritin, alpha-fetoprotein (AFP), soluble IL-2R alpha and cortisol levels. The etiology was considered unidentified when cases remained without a definitive diagnosis after extensive tests.

Patients with chronic liver disease presenting as NALF (NALF-CLD) were defined as those with liver failure who presented splenomegaly, ascites and congenital cirrhosis on ultrasound. In the absence of liver transplantation (LT), liver failure resolution was considered when the INR was < 1.4. In our laboratory, a coagulation test indicating "no clot formation" was defined as an INR > 15 (in this case, 15 was the assumed value for statistical analysis). The upper limits for the detection of AFP and ferritin were 121000 ng/mL and 2000 ng/mL, respectively (in these patients, 121000 and 2000 ng/mL were the assumed values for statistical analysis).

Infants were considered for LT and referred to a liver transplant center when the INR was persistently > 4 despite appropriate management. Contraindications to LT included hemophagocytic lymphohistiocytosis (HLH), active infection, mitochondrial disorders exhibiting rapid neurologic decline, multiple organ dysfunction syndrome (MODS).

We collected the following data from electronic medical records: demographic information, clinical manifestations, laboratory and imaging results, clinical interventions, and outcomes. All work conformed to the STROBE (Strengthening the Reporting of Observational Studies in Epidemiology) guidelines [10].

### Statistical analysis

The statistical analysis was performed using SPSS 22.0 statistical software (SPSS, Inc., Chicago, IL, USA) and MedCalc.lnk (MedCalc Software, Ostend, Belgium). Continuous variables are presented as medians with interquartile ranges (IQRs), and categorical variables are presented as percentages. Differences in laboratory values among patients with different etiologies were calculated by the Kruskal-Wallis test with Bonferroni post hoc correction for multiple comparisons. HLH was excluded from the above analysis due to the sample size of 1. The Mann-Whitney test and Fisher’s exact test were used to compare outcomes. The Kaplan-Meier method was applied to estimate the median survival times of the entire cohort, infection cohort and GALD-NH cohort. A *P* value < 0.05 was considered to indicate statistical significance.

## Results

### Etiology

**Fig. 1:**
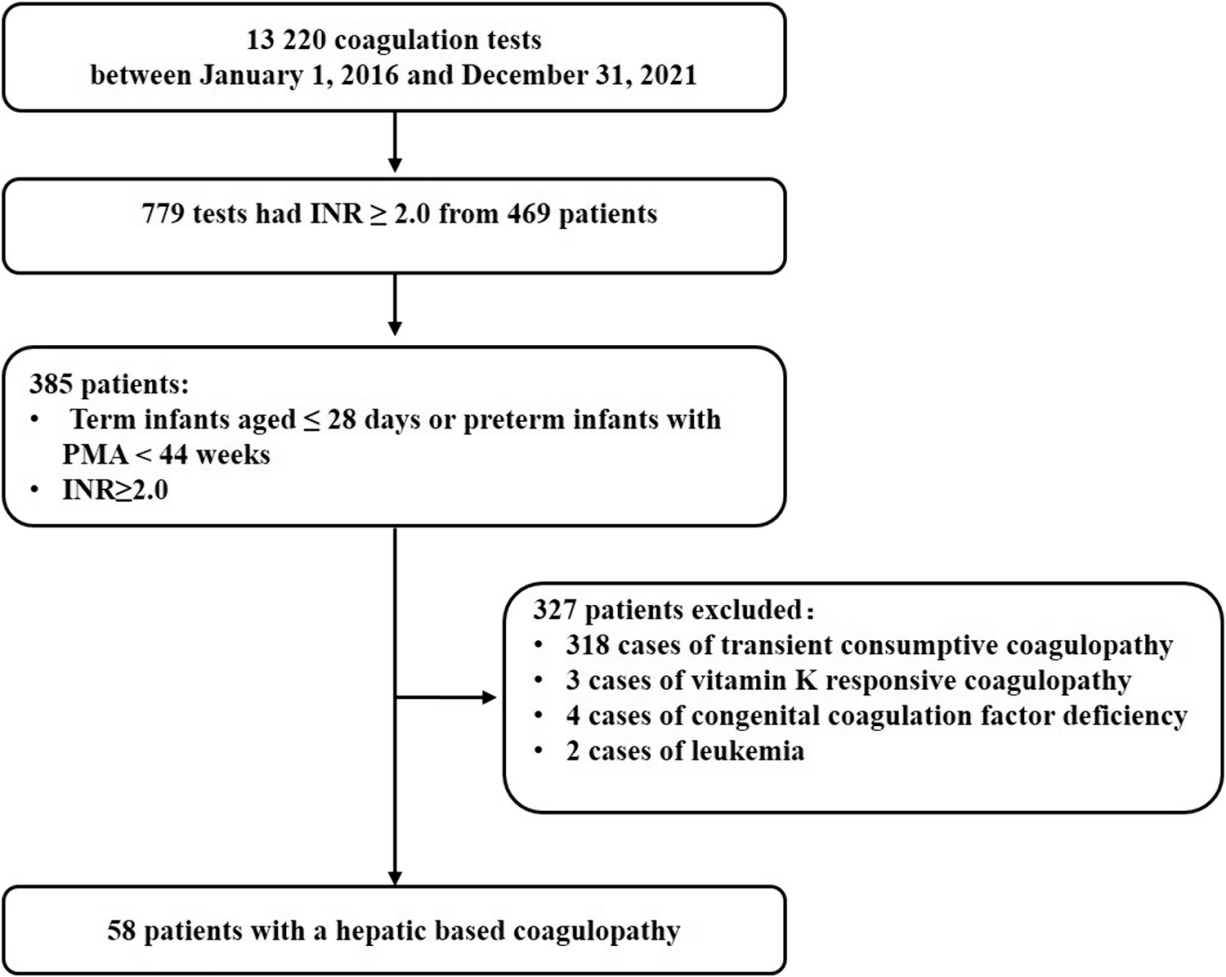
Case ascertainment in the neonatal acute liver failure group. *INR* International normalized ratio, *PMA* Postmenstrual age

Fifty-eight patients with NALF were ultimately included in this study (Fig. 1). The etiology of NALF was identified in 86.2% (50/58) of infants (Table 1). Hypoxic/ischemic injury was the most common identified etiology, occurring in 29.3% (17/58) of the patients, followed by infection (27.6%, 16/58) and GALD-NH (10.3%, 6/58) In the hypoxic/ischemic group, eleven (19%, 11/58) patients experienced severe perinatal hypoxia-ischemia, whereas the other six patients (10.3%, 6/58) experienced cardiac-associated ischemia or hypovolemic shock. The etiologies observed in the infection group included eight (13.8%, 8/58) cases of viral infection, five (8.6%, 5/58) cases of bacterial infection, two (3.4%, 2/58) cases of fungal infection and one (1.7%, 1/58) case of congenital tuberculosis. Enterovirus infection accounted for 87.5% (7/8) of the viral infections, and rubella virus infection accounted for the remaining 12.5% (1/8). The IMD group in our series included three patients: one with a urea cycle disorder, one with carnitine palmitoyltransferase II deficiency, and one with hereditary fructose intolerance. Among the patients with other etiologies, four had focal liver lesions, one had portal vein thrombosis, one had intrahepatic portosystemic shunts (IPSS), and one had adrenocortical insufficiency.

**Table 1:** Patients’ demographics and clinical features by etiology of neonatal acute liver failure (NALF)

Etiology, n	M:F	Preterm	Birth weight (g)	Age at diagnosis of ALF (days)	Problems during pregnancy	Family history	Bleeding	NALF-CLD	MODS
Hypoxic/ischemic (17)	7:10	41.2% (7/17)	2800 (2030–3465)	1 (1–3.5)	64.7% (11/17)	23.5% (4/17)	47.1% (8/17)	0%	82.4% (14/17)
Infection (16)	2:14	37.5% (6/16)	2610 (1985–3063.8)	7.5 (5–18.3)	43.8% (7/16)	6.25% (1/16)	31.3% (5/16)	0%	62.5% (10/16)
GALD-NH (6)	2:4	16.7% (1/6)	2800 (2570–3225)	5.5 (3–13)	0%	33.3% (2/6)	66.7% (4/6)	16.7% (1/6)	83.3% (5/6)
Inherited metabolic diseases (3)	1:2	0%	3220 (2800-)	2 (2-)	0%	66.7% (2/3)	66.7% (2/3)	0%	66.7% (2/3)
HLH (1)	1:0	0%	3600	14	0%	100% (1/1)	0%	0%	100% (1/1)
Other etilologies (7)	6:1	71.4% (5/7)	2280 (1425–3300)	3 (1–4)	57.1% (4/7)	28.6% (2/7)	57.1% (4/7)	0%	28.6% (2/7)
Unknown (8)	7:1	25% (2/8)	2825 (2350–3300)	4.5 (1.5–24.5)	12.5% (1/8)	25% (2/8)	37.5% (3/8)	50% (4/8)	50% (4/8)
Total (58)	26:32	36.2% (21/58)	2800 (2195–3300)	4 (1–13.3)	39.7% (23/58)	24.1% (14/58)	44.8% (26/58)	8.6% (5/58)	65.5% (38/58)

Data were expressed as median (interquartile range, IQR) or percentage

*CLD* chronic liver disease, *F* female, *HLH* hemophagocytic lymphohistiocytosis, *GALD-NH* gestational alloimmune liver disease with neonatal hemochromatosis, *M* male, *MODS* multiple organ dysfunction syndrome

In addition, the etiology could not be determined in 13.8% (8/58) of the patients despite extensive examinations including medical exome sequencing [11] or trio whole-exome sequencing (trio-WES). One girl developed severe hyperlactacidemia complicated with liver, heart, and kidney symptoms shortly after birth, so mitochondrial disease was suspected. However, she did not undergo further respiratory chain enzymes and mtDNA copy number tests of the liver and muscle due to her critical condition. 50% (4/8) cases of undetermined etiology had NALF-CLD but did not undergo liver biopsy due to critical condition or family member refusal.

### Clinical features and laboratory data

The demographics and clinical features of these patients is shown in Table 1. Median gestational age at birth was 38 weeks (IQR: 35–39 weeks), and 44.8% (26/58) were boys. Median age at diagnosis was 4 days (IQR: 1–13.3 days); 69% (40/58) were diagnosed within the first 7 days after birth and 27.6% (16/58) in the first day after birth. Three premature infants (all born at less than 29 weeks of gestational age) were diagnosed with ALF 28 days after birth (all diagnosed at less than 39 weeks of postmenstrual age). Patients with hypoxic/ischemic injury were diagnosed at the youngest age (median: 1 days, IQR: 1–3.5 days), and 64.7% (11/17) of them presented with fetal distress. Family history of repeated miscarriages, neonatal death or ALF in siblings was most common among HLH (100%, 1/1), IMD (66.7%, 2/3) and GALD-NH (33.3%, 2/6). Bleeding was a common complication (44.8%, 26/58) involving the gastrointestinal tract (27.6%, 16/58), lungs (12.1%, 7/58), intracranial space (10.3%, 6/58), skin (5.2%, 3/58), adrenal glands (3.4%, 2/58), and intraperitoneal cavity (1.7%, 1/58). Patients with other etiologies had the least MODS (28.6%, 2/7).

**Fig. 2:**
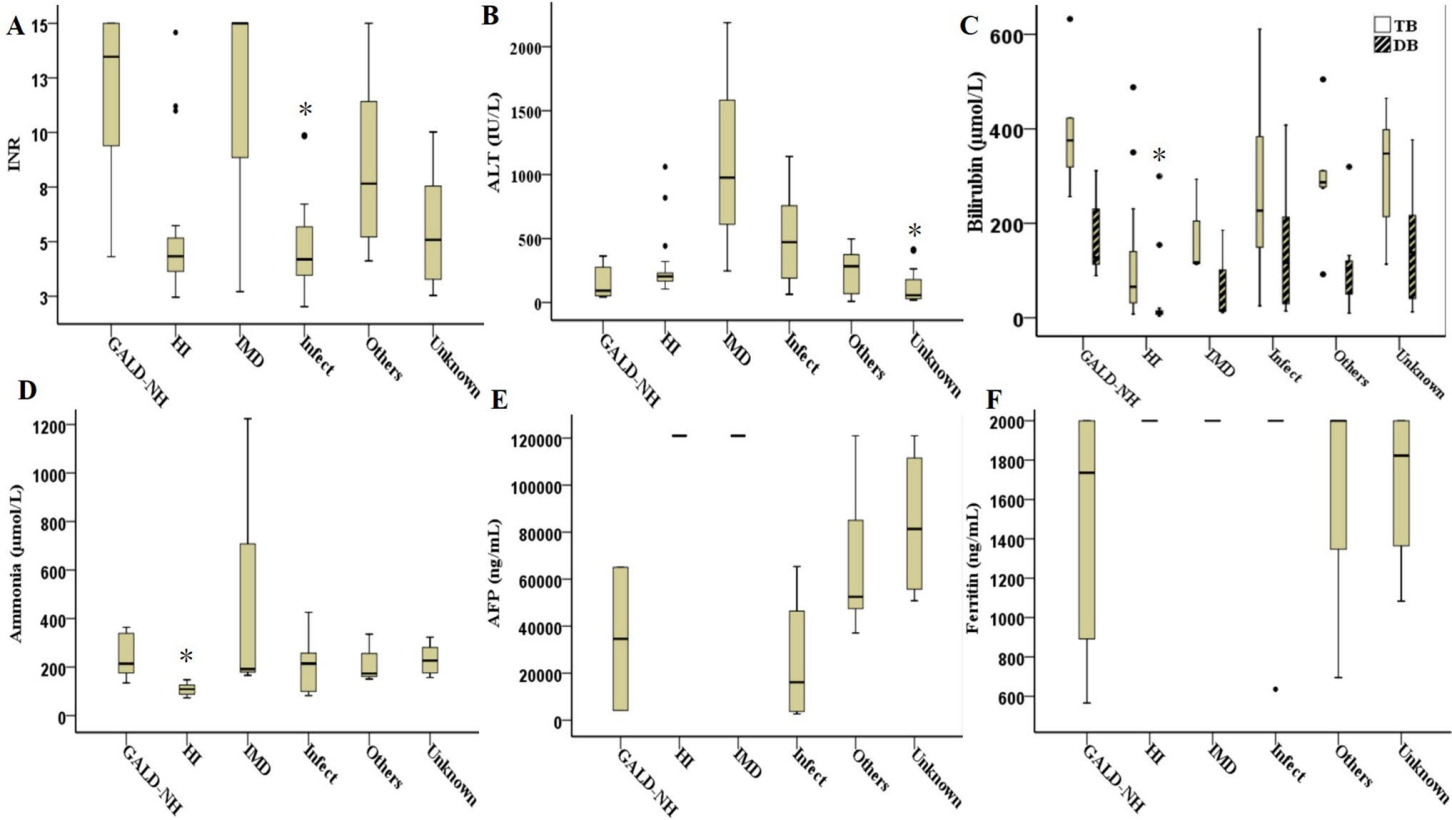
Peak laboratory values of infants with acute liver failure during neonatal hospitalization. **A** The INR in the infection group (4.2) was significantly lower than that in the GALD-NH group (13.5). **B** Patients with an unknown etiology had significantly lower ALT levels (57.1 IU/L) than did those with an IMD (977 IU/L) or infection (472.3 IU/L). **C** Compared with those in the GALD-NH and unknown cause groups, the TB and DB levels (65.6 and 11.1 μmol/L, respectively) were significantly lower in the HI group. **D** Ammonia levels in the HI group (109 μmol/L) were significantly lower than those in the GALD-NH and unknown cause groups (214 and 227 μmol/L, respectively). **E**, **F** There was no significant difference in AFP (*P* = 0.18) or ferritin (*P* = 0.3) levels among the different etiological groups. *AFP* Alpha-fetoprotein, *ALT* Alanine aminotransferase, *DB* Direct bilirubin, *GALD-NH* Gestational alloimmune liver disease with neonatal hemochromatosis, *HI* Hypoxic/ischemic injury, *IMD* Inherited metabolic diseases, *INR* International normalized ratio, *Infect* Infection, *TB* Total bilirubin. ^∗^*P* < 0.05 for multiple comparisons

Figure 2 shows the peaks of laboratory data during hospitalization for neonates with different etiologies. Ammonia was measured in 74.1% (43/58) of the patients, and 39.5% (17/43) had hyperammonemia (> 200 μmol/L). A total of 31% of the patients (18/58) had a recorded AFP value, and 46.6% (27/58) had a recorded ferritin value. The distributions of the INR and alanine aminotransferase, total bilirubin, direct bilirubin (DB) and ammonia levels differed for each etiological group, and the differences were statistically significant (all p values less than 0.05) (Fig. 2A–D). No significant difference was found in the AFP value (*P* = 0.18) or in the ferritin level (*P* = 0.3) among the different etiological groups (Fig. 2E, F).

### Treatment and outcomes

Only 6.9% (4/58) of the patients, including two with viral infection, received empiric acyclovir after symptom onset. All infants with GALD-NH received intravenous immunoglobulin (IVIG) beginning at the onset of illness, but none of the patients received double volume exchange transfusion due to improvement, parental refusal or death. Two infants were listed for LT, but neither received a LT due to parental refusal, and one (with GALD-NH) ultimately died of multiple organ failure and the other (with IPSS) was automatically discharged from the hospital and lost to follow-up.

**Fig. 3:**
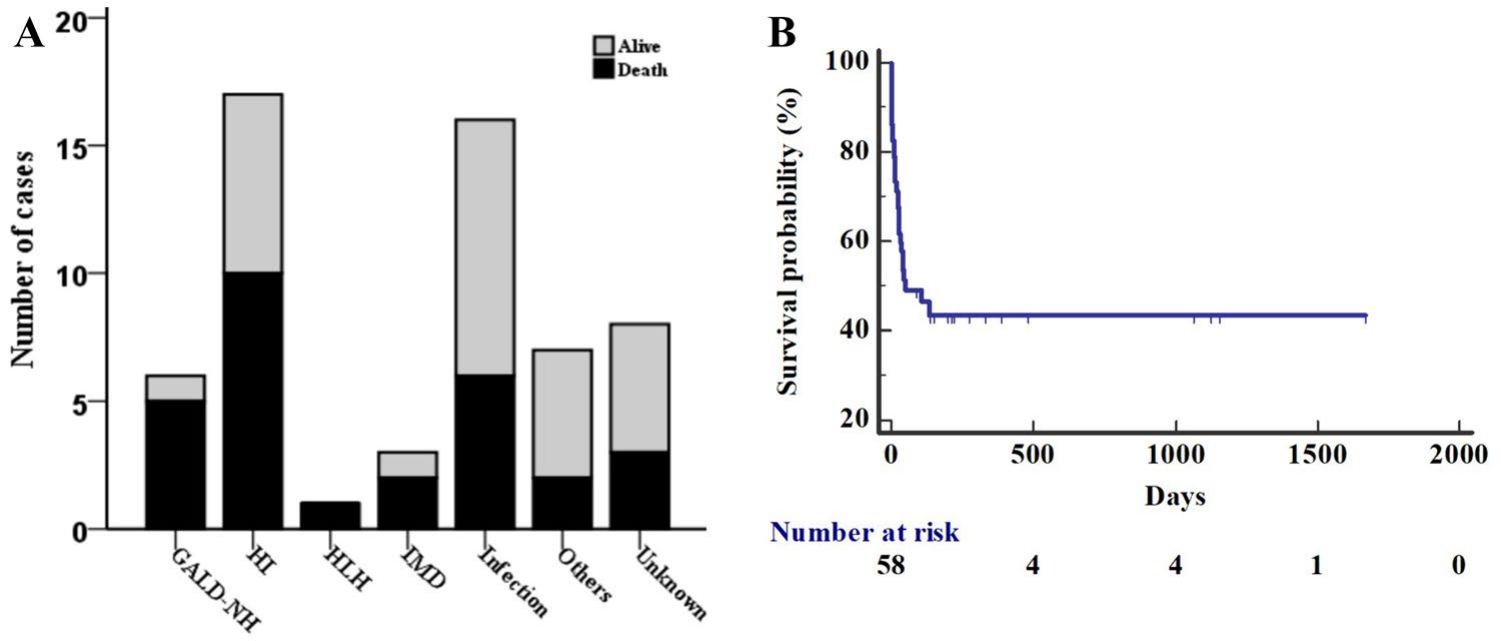
Outcomes of neonatal acute liver failure patients. **A** The mortality rate was greater than 50% in the GALD-NH (83.3%, 5/6), HLH (100%, 1/1), IMD (66.7%, 2/3) and HI (58.8%, 10/17) groups. **B** The cumulative median survival time for the whole cohort was 48 days (95% CI: 0.0–140.7 days). *GALD-NH* Gestational alloimmune liver disease with neonatal hemochromatosis, *HI* Hypoxic/ischemic injury, *HLH* Hemophagocytic lymphohistiocytosis, *IMD* Inherited metabolic diseases

At the last follow-up, liver function had completely recovered in eighteen patients (31%, 18/58) (median duration of liver failure: 10.5 days, IQR: 3.5–17.5 days), and all of those patients survived, 50% (9/18) of whom were in the infection group. The overall mortality rate was 50% (29/58) at the last contact with all survivors (median age: 34.5 days, IQR: 7–141 days) (Fig. 3A). Most of the deaths were from hypoxic/ischemic (34.5%, 10/29), infection (20.7%, 6/29) and GALD-NH (17.2%, 5/29) groups. The cumulative median survival time for the whole cohort was 48 days (95% CI: 0.0–140.7 days) (Fig. 3B), with a median survival time of 26 days (95% CI: 0.0–62.9 days) for the hypoxic/ischemic injury group and 43 days (95% CI: 0.0–125.8 days) for the GALD-NH group.

The DB level in the patients who survived was significantly greater than that in the patients who died (*P* = 0.025), and the incidence of cholestatic liver injury in the surviving patients (69%, 20/29) was significantly greater than that in the deceased patients (38%, 11/29) (*P* = 0.018). Other characteristics were not significantly different between patients who survived and patients who died (Table 2).

**Table 2:** Patients features of different outcomes

	Survival (IQR; n)	Death (IQR; n)	*P* value
Birth weight (g)	2850 (2375–3300; 29)	2650 (2030–3235; 29)	0.379
Gestational age (weeks)	38.4 (35.6–39.2; 29)	37.4 (33.9–38.3; 29)	0.146
Age at diagnosis (days)	4 (2–10.5; 29)	5 (1–14; 29)	0.912
INR	4.3 (3.6–6.6; 29)	5.7 (3.9–10.8; 29)	0.118
ALT (IU/L)	242 (102.3–497.9; 29)	203.9 (112.8–324; 29)	0.423
DB (μmol/L)	108.9 (26.5–214.5; 29)	20.4 (11–140.6)	0.025
Ammonia (μmol/L)	166 (107.1–224; 23)	204 (132.2–308.3; 20)	0.113
AFP (ng/mL)	60651 (42324–121000; 13)	4820.9 (2267.4–75051; 5)	0.125
Ferritin (ng/mL)	2000 (1223.8–2000; 16)	2000 (1741–2000; 11)	0.582

Of peak laboratory values during hospitalization, DB was significantly different between surviving and deceased patients (*P* = 0.025)

*AFP* Alpha-fetoprotein, *ALT* Alanine aminotransferase, *DB* Direct bilirubin, *INR* International normalized ratio, *TB* Total bilirubin

## Discussion

NALF is a rare and fatal disease; however, it is generally reported as poor, particularly in the Chinese population. This study was conducted at a large tertiary pediatric hospital in China. Our data showed hypoxic/ischemic injury, especially perinatal hypoxia-ischemia, predominated among the etiologies of NALF, which was consistent with the findings of Zozaya et al. [7]. Infection, the second most common etiology in our series, included not only viral infections, which have been highlighted in previous studies [6, 8], but also bacterial and fungal infections. We agree with Zozaya et al. [7] that a case of NALF can be considered secondary to septicemia only if the Pediatric Acute Liver Failure Study Group criteria are fully met when the pathogen is isolated from a blood culture sample. Surprisingly, herpes simplex virus, the most common virus that causes NALF [6, 8], was not found in our series; our results showed that the most common virus was enterovirus.

According to the published literature, the indeterminate etiology rate among neonates is approximately 5%–32% [6–9]. In our study, 13.8% of the patients had an unidentified cause. The primary pathogenic mechanism, such as hypoxic/ischemic injury or infection, can be quickly diagnosed by recognizable clinical scenarios and laboratory test results. NH, mainly caused by GALD, can be diagnosed by positive MRI or oral mucosal biopsy findings of extrahepatic siderosis, but a negative finding cannot exclude GALD [12, 13]. The definitive confirmation of GALD is positive C5b-9 staining by liver biopsy; however, its use as a routine diagnostic tool is unfeasible. Compared with ALF, metabolic disorders rarely cause NALF. However, this may not actually be the case. Metabolic diseases are a major category of disease and can be easily diagnosed through metabolic screening and next-generation sequencing; however, others diseases (such as mitochondrial disorders) require more invasive operations for definitive diagnosis, such as muscle or liver biopsy [14]. In our study, medical exome sequencing [11] or trio-WES was performed for neonates with a suspected genetic metabolic disease or an unknown causes of ALF, resulting in the diagnosis of three patients. We suspected that a girl with severe hyperlactacidemia might have mitochondrial disease, but did not perform further mDNA sequencing or biopsy of clinically relevant tissue because of her critical condition. Despite advances in diagnostic techniques, an extensive diagnostic work-up is not easily performed for critically ill newborns. Storing blood, urine, and liver tissue samples for future studies is strongly recommended for patients with unknown causes of ALF.

The fetal-neonatal continuum of liver disease is defined by the fact that some causes of neonatal liver failure actually begin with fetal liver disease [3]. As early as 2001, Jackson et al. [15] proposed that neonatal liver failure (liver failure at 60 days of age, according to their definition) be categorized as “acute hepatocellular necrosis” or “chronic liver disease (CLD)” (that is, the extension of fetal liver disease). The Clinical Practice Guidelines of the Italian Society of Pediatric Gastroenterology, Hepatology and Nutrition suggest that pediatric ALF be subclassified into “pure” forms of ALF and CLD presenting with a phenotype of ALF [1]. In our study, five infants developed splenomegaly, ascites and cirrhosis on ultrasound in the neonatal period, suggesting chronic liver injury. GALD predominated in the NALF-CLD group [3, 15]. Of the five patients in our study, only one was diagnosed with NH-associated GALD, but the remaining four patients had unknown etiologies and did not undergo further liver biopsy during follow-up due to critical conditions or family member refusal.

Despite prompt medical therapy, the survival with native liver (SNL) rate in our study was low (50%), consistent with previous reports of approximately 33% to 47.8% [6–9]. LT is the only option for treating ALF when standard medical therapy fails [16]; however, no one received a LT in our cohort. The main reason for this may be the high incidence of active infections and MODS, which are contraindications to LT, partly due to family refusal. Antala et al. [9] conducted a multicenter retrospective study on peritransplant outcomes in NALF patients and reported a lower rate of LT (2.0% vs. 6.4%; *P* < 0.001) in these patients than in older infants (31–120 days old). However, risk factors for death or transplant and posttransplant outcomes were similar between neonates and older infants. Therefore, further studies are needed to better optimize decision algorithms for LT in NALF patients and improve outcomes [9, 16].

There are no reliable biochemical markers for predicting SNL. Zozaya et al. [7] demonstrated that higher ALT levels and INR values at diagnosis could predict poor prognosis in the short term. Borovsky et al. [6] reported that only a higher AFP level was present in SNL patients. However, our study showed that the incidence of cholestasis and the DB level were significantly greater in SNL patients. This result may be explained by the fact that cases secondary to severe perinatal asphyxia or cardiogenic/hypovolemic shock, which accounted for the majority of the deaths, had the lowest DB levels compared to the other groups Therefore, we agree with Squires et al. [2] that the etiology of ALF determines the short-term outcome. The prognosis of GALD-NH remained poor even in those patients who underwent LT. Our results showed a higher mortality rate than previously reported [6, 7, 17]. The reason for this discrepancy may be that all GALD-NH patients in our study received IVIG therapy, but none received double volume exchange transfusions or LT. Identifying the etiology of ALF is crucial for quickly instituting disease-specific therapies for treatable disorders and selecting patients who may benefit from LT.

One limitation of our study is that it was a single-center study with a small sample size due to the rarity of the disease and the diversity of etiologies, which led to predictive analysis in the absence of a larger cohort. However, multicenter studies are needed to address the sample size limitation and differences in results due to regional specificity.

Overall, we demonstrated that hypoxic/ischemic injury and infection are the predominant causes of NALF in Chinese population and can be easily distinguished by unique clinical and laboratory profiles. The population had an overall low SNL rate, and a proportion of the patients did not have a clear etiology. However, further research is needed to maximize the accuracy and accessibility of diagnostic tests and minimize their invasiveness to achieve accurate and timely diagnosis and improved outcomes.

## Data Availability

No datasets were generated or analysed during the current study.

## Abbreviations

AFP: Alpha-fetoprotein
ALF: Acute liver failure
CLD: Chronic liver disease
DB: Direct bilirubin
GALD-NH: Gestational alloimmune liver disease with neonatal hemochromatosis
HLH: Hemophagocytic lymphohistiocytosis
LT: Liver transplantation
IMD: Inherited metabolic diseases
INR: International normalized ratio
IQR: Interquartile range
IPSS: Intrahepatic portosystemic shunts
IVIG: Intravenous immunoglobulin
MODS: Multiple organ dysfunction syndrome
MRI: Magnetic resonance imaging
MtDNA: Mitochondrial DNA
NALF: Neonatal acute liver failure
SNL: Survival with native liver
WES: Whole-exome sequencing
